# Organ development in growth‐restricted fetuses in the reduced uterine perfusion pressure rat model: A vascular approach of brain, heart, and kidney

**DOI:** 10.14814/phy2.70244

**Published:** 2025-02-12

**Authors:** J. Alhama‐Riba, C. M. van Kammen, K. T. Nijholt, D. Viveen, K. Amarouchi, D. Shasha, M. M. Krebber, F. E. Hoebeek, A. T. Lely, C. H. A. Nijboer, F. Terstappen

**Affiliations:** ^1^ Division of Woman & Baby, Department for Developmental Origins of Disease (DDOD), Brain Center UMC, Wilhelmina Children's Hospital Utrecht University Utrecht the Netherlands; ^2^ Division of LAB, Department CDL Research, Nanomedicine University Medical Center Utrecht, Utrecht University Utrecht the Netherlands; ^3^ Division of Woman & Baby, Department of Obstetrics, Wilhelmina Children's Hospital Utrecht University Utrecht the Netherlands; ^4^ Division of Internal Medicine and Dermatology, Department of Nephrology and Hypertension University Medical Center Utrecht the Netherlands; ^5^ Division of Woman & Baby, Department of Neonatology, Wilhelmina Children's Hospital Utrecht University Utrecht the Netherlands

**Keywords:** brain, fetal growth restriction, heart, kidney, vascular

## Abstract

Fetal growth restriction (FGR) increases the risk of developing cardiovascular, renal, and neurovascular diseases. An overlapping vascular pathophysiology as a response to chronic hypoxia and circulatory redistribution in utero, might underlie this lifelong burden. This study aims to assess potential vascular detoriations in multiple organs following FGR using the Reduced Uterine Perfusion Pressure (RUPP) rat model. The fetal brain, heart, and kidney were collected (RUPP *n* = 16 vs. sham *n* = 13) at embryonic day (E)19 for histological assessment of various aspects of vascular and structural development. Results indicated similar microvascularisation in all organs between the groups. Structural assessment demonstrated a decreased brain area and thickness of the somatosensory cortex and thicker right ventricular wall of the heart (not driven by increased proliferation) in RUPP fetuses, and no differences in renal development. In conclusion, the fetal stage might be too early to detect detoriation in organ vasculature, while this study did reveal subtle alterations in structural development of mostly the brain, followed by the heart with sparing of the kidneys. Potentially compensatory mechanisms may be at play at this fetal stage. Nevertheless, small subclinical adaptations could make the FGR offspring more susceptible for second hits with manifestation at older age.

## 1 INTRODUCTION

Fetal growth restriction (FGR) leads to increased susceptibility to develop cardiovascular, renal, and neurovascular disease later in life, including hypertension, chronic kidney disease, and stroke (Barker, 2006; Calkins & Devaskar, 2011; Malhotra et al., 2019). The ‘Developmental Origin of Disease’ hypothesis states that the predisposition of non‐communicable disease originates in the womb, highlighting the crucial period of organogenesis and epigenetic fetal programming (Barker, 2006).

A dysfunctional placenta forms the most important cause of FGR in which an inadequate supply of oxygen and nutrients toward the fetus negatively influences fetal growth and organ development (Malhotra et al., 2019; Nardozza et al., 2017). The chronically hypoxic placenta also leads to hemodynamic redistribution toward fetal brain and heart at the expense of other organs such as the kidney (Giussani, 2016; Miller et al., 2016). While these compensatory mechanisms improve perinatal survival of growth‐restricted fetuses, the altered (both increased or decreased) organ perfusion is considered to be unfavorable in adulthood (Figueras et al., 2011; Malhotra et al., 2019).

However, how these changes in fetal organ perfusion and oxygenation contribute to a higher risk of developing diseases in multiple organs in FGR later in life, remains unexplored territory (Sehgal, 2011; Visentin et al., 2014). Animal studies show that fetal exposure to hypoxia changes the neurovascular unit (NVU), which hampers blood–brain‐barrier functionality through an increased permeability (Herrera & González‐Candia, 2021; Wu et al., 2024a). Cardiovascular adaptation presents mostly as endothelial‐dependent vascular stiffness and hypertension leading to cardiac remodeling in FGR; however the vasculature of the heart itself has not been studied yet (Alexander, 2003; Crepel et al., 1988; Rock et al., 2023). Fetal hypoxia has also been shown to impact kidney glomerular endothelial cell functionality by decreasing podocyte‐endothelial binding, resulting in endothelial‐dependent hypertension (Alexander, 2003; Zohdi et al., 2012). At a cellular level, it has been demonstrated that a (chronic) hypoxic period induces hypoxia‐induced factor (HIF) and vascular endothelial growth factor (VEGF) and impedes nitric oxide synthase (eNOS) in fetal endothelial cells, eventually causing endothelial dysfunction, vascular remodeling and increased vessel permeability in fetal organs, such as the brain, heart and kidney (Visentin et al., 2014).

This study aims to investigate whether vascular and structural alterations are present in multiple organs after exposure to placental insufficiency in growth‐restricted fetuses. We examined potential histological changes in the fetal brain, heart, and kidney in the ‘Reduced Uterine Perfusion Pressure’ (RUPP) model for placental insufficiency‐induced FGR in rats (Kammen et al., 2024; Granger et al., 2006; Li et al., 2012). The relatively early onset of placental insufficiency during pregnancy makes the RUPP model suitable for our multi‐organ approach since fetal organ development (including nephrogenesis) is still ongoing in the third trimester. The possible vascular overlapping mechanism could eventually offer potential targets for prevention or intervention for lifelong gain on cerebrovascular, cardiovascular, and renal health in FGR offspring.

## 2 MATERIALS AND METHODS

### 2.1 Animals

Twenty‐nine timed‐paired virgin pregnant Sprague–Dawley (SD) rats (225 g; 11–12 weeks) were purchased from Envigo, Horst, the Netherlands, on embryonic (E) days 5 and 6 (E5/E6). The rats were habituated at the animal facility (Utrecht University) prior to the start of the experiment at E14. The animals were housed in pairs or groups of three in conventional cages (Eurostandard Type IV S, 210 mm height × 375 mm width × 480 mm depth, West Chester, PA, USA) with woodchip bedding and enriched with a plastic shelter, wooden chew sticks and paper tissues, under a 12 h light/dark cycle (lights on from 7:00–19:00), humidity of 45%–65%, temperature between 20 and 24°C, and with free access to water and normal chow (rat/mouse maintenance, V1535‐000, Ssniff Spezialdiäten GmbH, Soest, Germany). The animal experiments were carried out in accordance with the Dutch and European guidelines. They were approved by the experimental animal committee Utrecht (Utrecht University, Utrecht, the Netherlands) and the central authority for scientific procedures on animals (The Hague, the Netherlands) (project identification code: AVD11500202010346, protocol 10,346–1–01, approved on June 8th 2022), and reported according to the ARRIVE guidelines (checklist added in supplemental data Table S1).

### 2.2 RUPP surgery to induce FGR in rats

We used the RUPP model of placental insufficiency‐induced FGR and preeclampsia (Kammen et al., 2024; Granger et al., 2006; Li et al., 2012). The dams were randomly allocated to the RUPP (*n* = 16) or sham (*n* = 13) procedure at E14; per cage, the first dam was allocated to the RUPP procedure and the second dam as a sham, and in case of three dams in the cage the middle dam was allocated to RUPP as well. The rats received Carprofen (5 mg/kg of body weight) subcutaneously at least 30 min before and 24 h after the surgical procedure. A laparotomy via a midline incision was performed under isoflurane anesthesia (ISOFLO® 100% W/W Liquid for inhalation vapor, Zoetis B.V., Rotterdam, the Netherlands; 4%–5% at induction and 2%–3% during the procedure) to unfold the uterine horns. The number of viable and non‐viable (resorbed) fetuses was counted. Clips were made from silver foil with 0.25 mm thickness and 99.95% purity (AG00‐FL‐000450, Goodfellow Cambridge Ltd., Huntingdon, UK). One clip (length 1.5 cm, width 1.5 mm, inner diameter 0.2 mm) was placed around the lower abdominal aorta above the iliac bifurcation. In addition, bilaterally a clip (length 1.0 cm, width 1.5 cm, inner diameter 0.1 mm) was placed around the left and the right ovarian arteries to reduce compensatory blood flow. The uterine horns were placed back into the abdomen and muscle and the skin layers were separately sutured (VICRYL® polyglactin 910 suture 4–0, V451H, Johnson & Johnson, Ohio, OH, USA). The sham procedure followed the same surgical steps, but the clips were placed randomly in fat tissue surrounding the uterus or ovaries instead of around vessels. After the surgical procedure, all rats were housed individually until sacrifice and checked regularly to ensure recovery. A priori exclusion of the dams (of either group) was applied when the surgical procedure resulted in total resorptions at E19. Considering the visible differences between RUPP and sham (maternal weight loss and higher animal welfare scores, namely more severe symptoms, after RUPP surgery) the researchers were not fully blinded during the tissue collection and blood pressure (BP) measuring phase of the experiment.

### 2.3 Urine collection and analysis of proteinuria

At E17 we collected urine samples by individually housing the dams in metabolic cages for 24 h in the same room under the same conditions as previously described with ad libitum access to food and water. The urine samples were centrifuged at 3000 rpm at 4°C for 10 min and saved at −80°C until analysis. Proteinuria was measured by the Bradford protein assay (Bio‐Rad Laboratories B.V., Veenendaal, the Netherlands). Proteinuria was defined as >20 mg/24 h.

### 2.4 Carotid artery cannulation

At E18 the dams underwent carotid artery cannulation to measure maternal BP to confirm that the RUPP model induces a preeclamptic phenotype. The rats received carprofen (5 mg/kg of body weight, s.c.) as analgesia at least 30 min prior and 24 h after the surgical procedure. The procedure was performed under isoflurane anesthesia (4%–5% at induction and 2%–3% during the procedure). An incision on the ventral side of the neck exposed the left carotid artery. A small cut by micro‐scissors in the artery between a knot at the cephalic end and a vessel clamp at the caudal end of the artery allowed cannulation with catheters prefilled with heparin‐saline (300 mg/mL, Heparin LEO 5.000 IU, LEO Pharma B.V., Amsterdam, the Netherlands). The catheter was tunneled subcutaneously and exteriorized between the shoulder blades. The incision in the neck was closed using surgical tissue adhesive (3 M™ Vetbond Tissue Adhesive 1469, 3 M, Saint Paul, MN, USA). The handmade catheters contained small gauge tubing (inner diameter of 0.28 mm and outer diameter of 0.64 mm, Medical Grade Micro Vinyl Catheter Tubing, BB31785‐V/1, Scientific Commodities Inc., Lake Havasu City, AZ, USA) inserting the vessel and medium gauge tubing (inner diameter of 0.58 mm and outer diameter of 0.99 mm, V3 tubing, Scientific Commodities Inc., Lake Havasu City, AZ, USA) at the other end available for BP measurements. The catheters were sterilized before use in a sterile bag under UV radiation for 24 h in a laminar flow cabinet.

### 2.5 Recording and analysis of maternal BP

During the habituation period, we placed the dams in restrainers three times prior to the actual BP measurements. The maternal BP and heart rate (HR) were recorded in conscious rats at E19 (LabChart Pro v8.1.19, ADInstruments, Dunedin, New Zealand). The carotid artery catheters were connected to the pressure transducer. The signal was amplified (PowerLab 8/35, ADInstruments, Dunedin, New Zealand), digitized and recorded with LabChart Pro v8.1.19 (ADInstruments, Dunedin, New Zealand). To ensure stress reduction from handling and fixation, we implemented a habituation period of at least 30 min before measuring BP and HR for a duration of at least 45 min. Artifacts (HR values ≤300 bpm and ≥ 600 bpm) were removed from the analysis.

### 2.6 Tissue collection and processing

The dams were euthanized under isoflurane anesthesia via total bleeding at E19. At least 30 min before opening the abdominal cavity, the rats received carprofen (5 mg/kg body weight, subcutaneously) as an analgesic. Subsequently, the abdominal cavity was opened, and the uterine horns were exposed. We counted the number of viable and non‐viable fetuses (resorbed), which were then removed from the uterine horns. The individual fetal and placental weights, and fetal crown‐rump lengths, were measured and averaged per litter. Additionally, the fetal‐placental weight ratios (FW) were calculated for each unit and averaged per litter. Brain, kidneys, and heart from one fetus per litter were randomly collected from one of the uterine horns, as the dam was considered the experimental unit. The right kidney was snap‐frozen in liquid nitrogen, stored at −80°C, and later used for sex determination. The fetal head (skull and brain), left kidney, and heart were placed in 4% PFA in PBS for 24–48 h at 4°C. Afterwards, the brain was removed from the skull. Subsequently, the organs were placed in increasing ethanol concentrations (30%, 50% and 70%) and then further dehydrated to 100% ethanol and submerged into paraffin in the Microm STP120 Spin Tissue Processor (Thermo Fisher Scientific, Waltham, MA, USA), according to standard protocol. One kidney (sham) and one heart (RUPP) were lost during the embedding procedure. An overview of the blinding and the exclusions was added in Table S2.

To determine sex differences, DNA was isolated from snap‐frozen right kidney samples. Samples were incubated in a cell lysis and proteinase K solution (1 M Tris–HCl (pH 8.5), 5 M NaCl, 0.5 M EDTA (pH 8.0), 20% SDS (1,610,418, Bio‐rad, Bio‐Rad Laboratories B.V., Veenendaal, the Netherlands), sterile H_2_O_2_, proteinase K (10 mg/mL; 3,115,836,001, Roche, Basel, Switzerland)) for 30 min at 55°C. Proteinase K was inactivated at 100°C for 5 min, and samples were spun at 4°C (13,300 rpm) for 4 min. After collecting the supernatant, an equal volume of cold isopropanol (VWR, Radnor, PA, USA) was added, and DNA was precipitated by spinning at 4°C for 15 min (13,300 rpm). The DNA pellet was washed with 70% ethanol, vortexed, spun, and dissolved in Tris‐EDTA buffer. DNA was rehydrated for 15 min at 65°C, spun, and quantified using a Nanodrop 2000 Spectrophotometer (Thermo Fisher Scientific, Waltham, MA, USA). The single‐step PCR method with three primers described by Dhakal & Soares (Merck, Darmstadt, Germany) (Dhakal & Soares, 2017) was then used. Each primer was diluted to 10 μM using nuclease free water (Fresenius Kabi Nederland, Huis ter Heide, the Netherlands). Each PCR reaction contained 25 μL of 2X DreamTaq Green PCR Master Mix (K1081, Thermo Fisher Scientific, Waltham, MA, USA), 21 μL of nuclease‐free water (Fresenius Kabi Nederland, Huis ter Heide, the Netherlands), 1 μL of each primer (10 μM) and 1 μL of DNA template (250 ng/μL). PCR was performed with 40 cycles (95°C for 30s, 59°C for 30s, 72°C for 1 min) and a final extension at 72°C for 7 min using a T100 thermo cycler (Bio‐rad, Bio‐Rad Laboratories B.V., Veenendaal, the Netherlands). PCR products (15 μL) were run on a 2% agarose gel (Roche, Basel, Switzerland) in a 1X TAE buffer at 80 V for 30 min, and imaged using an ImageQuant 800 under UV light (Cytiva, Marlborough, MA, USA).

### 2.7 Immunohistochemistry

#### 2.7.1 Brain

##### Histological techniques and analysis

Brains were cut in coronal sections at 8 μm thickness at the coronal level corresponding to Figure of the Paxinos & Ashwell Atlas of the Developing Rat Nervous System, Edition 4 (E19 coronal 24) (Paxinos & Ashwell, 2018). Brain slices were stained with hematoxylin and eosin (H&E), for the visualization of cells, to assess cortical thickness at three different cortical regions: 1. A30 as part of the retrosplenial cortex, 2. motor cortex, and 3. somatosensory cortical region. To assess brain area and cortical thickness, full‐section images were taken with a Nikon D1 digital camera (Nikon, Tokyo, Japan) and were analyzed using Fiji software v1.53 (National Institutes of Health, NIH, Bethesda, MD, USA). For brain area measurements, images were converted to 8‐bit and manually thresholded. The thickness of A30, motor, and somatosensory cortical regions was measured per region and hemisphere. Results shown the average of both hemispheres per analyzed cortical region. To quantify the number of cerebral microbleeds, images were taken at 12 different locations throughout the brain section with an Axio Imager light microscope with Zen software and equipped with an AxioCam ICc 5 (Carl Zeiss, Oberkochen, Germany) at 10× magnification (Objective N‐Achroplan 10×/0.25 M27, 420,940–9901, Carl Zeiss, Oberkochen, Germany). The number of cerebral microbleeds observed in the 12 images was summed. Results show the total amount of cerebral microbleeds per animal normalized by brain area.

##### Immunohistochemistry and analysis

###### Laminin staining (microvasculature marker)

For laminin immunohistochemistry in brain sections, an adapted protocol from Castillo‐Melendez et al. (Castillo‐Melendez et al., 2017) was used. The brain sections were first deparaffinized using xylene, followed by a 100% ethanol step. Subsequently, the sections were rehydrated in 0.1 M PBS with 1% Triton‐X 100 for 15 min. Antigen retrieval was carried out by incubating the sections in 10 mM citrate buffer (pH 6) at 95°C for 15 min, followed by cooling on ice for 30 min. To block endogenous peroxidase activity, the sections were rinsed in PBS and incubated in 0.3% H_2_O_2_ and 50% methanol at room temperature for 15 min. Non‐specific binding was blocked by incubating the sections in 5% normal goat serum in 0.1 M PBS at room temperature for 30 min. The sections were then incubated overnight at 4°C with anti‐laminin rabbit polyclonal antibody (1:100; L9393, Merck, Darmstadt, Germany) in 2% normal goat serum. After washing in PBS, the sections were incubated with a secondary biotinylated goat anti‐rabbit antibody (1:100; BA‐1000, Vector Laboratories, Newark, CA, USA) at 37°C for 45 min, followed by another washing step in PBS. The antibody‐specific staining was enhanced using the Vectastain® ABC‐HRP kit (PK‐4000, Vector Laboratories, Newark, CA, USA) and visualized using 3,3’‐Diaminobenzidine tetrahydrochloride hydrate (DAB; J62216, Thermo Fisher Scientific, Waltham, MA, USA). Finally, the sections were mounted with DEPEX (18,243, Serva Electrophoresis GmbH, Heidelberg, Germany) and cover glass.

To quantify the laminin coverage, images were taken at 8 different locations throughout the brain section with an Axio Imager light microscope with Zen software and equipped with an AxioCam ICc 5 (Carl Zeiss, Oberkochen, Germany) at 10× magnification (Objective N‐Achroplan 10×/0.25 M27, 420,940–9901, Carl Zeiss, Oberkochen, Germany). The total number of laminin‐stained pixels was calculated per image using a personalized macro created in Fiji software v1.53 (National Institutes of Health, NIH, Bethesda, MD, USA). Results show the average of laminin coverage in the 8 different images normalized by brain area.

###### Albumin staining (Blood Brain Barrier [BBB] leakage marker)

For albumin immunohistochemistry, an adapted protocol from Gussenhoven et al. (Gussenhoven et al., 2019) was used. Brain sections were deparaffinized using xylene and rehydrated in decreasing concentrations of ethanol. Endogenous peroxidase activity was blocked by incubating the sections in 3% H_2_O_2_ in methanol for 20 min at room temperature, followed by washing with PBS. Antigen retrieval was performed in the same manner as for laminin staining. To block non‐specific binding, sections were incubated in 2% skim milk powder (Merck, Darmstadt, Germany) in PBS at room temperature for 30 min. The sections were then washed in PBS and incubated overnight at 4°C with anti‐albumin sheep polyclonal antibody (1:30,000; 0220–2424, Bio‐Rad Laboratories B.V., Veenendaal, the Netherlands) in PBS. After another washing step in PBS, the sections were incubated with a secondary peroxidase AffiniPure™ donkey anti‐sheep antibody (1:1000; 713–035‐147, Jackson ImmunoResearch Laboratories) in 0.25% PBS‐Triton® X‐100 (Merck, Darmstadt, Germany) at room temperature for 1 h, followed by washing in PBS. The antibody‐specific staining was enhanced using the Vectastain® ABC‐HRP kit (PK‐4000, Vector Laboratories, Newark, CA, USA) and visualized using 3,3’‐Diaminobenzidine tetrahydrochloride hydrate (DAB; J62216, Thermo Fisher Scientific, Waltham, MA, USA). The sections were then mounted with DEPEX (18,243, Serva Electrophoresis GmbH, Heidelberg, Germany) and cover glass.

For the assessment of BBB leakage, two adjacent coronal sections were used and four 2.5× images were taken per coronal section. Images were taken with an Axio Imager light microscope with Zen software and equipped with an AxioCam ICc 5 (Carl Zeiss, Oberkochen, Germany) at 2.5× magnification (Objective N‐Achroplan 2.5×/0.07 M27, 420,920–9901, Carl Zeiss, Oberkochen, Germany). When more detail was needed because of possible microbleeds, a higher magnification of 10× (Objective N‐Achroplan 10×/0.25 M27, 420,940–9901, Carl Zeiss, Oberkochen, Germany) was used. Results show the total amount of albumin leakages per animal normalized by brain area.

#### 2.7.2 Heart

##### Histological techniques and analysis

The heart was sectioned at the midventricular level in the transversal plane at 8 μm thickness and stained with H&E to assess the left and right ventricular (LV and RV) wall thickness at the macroscopic level and the number of nuclei at the microscopic level. For imaging, we used an Axio Imager light microscope with Zen software and equipped with an AxioCam ICc 5 (Carl Zeiss, Oberkochen, Germany). For the macroscopic analysis, one image at 10× magnification was used per animal (Objective N‐Achroplan 10×/0.25 M27, 420,940–9901, Carl Zeiss, Oberkochen, Germany). The wall thickness analysis consists of the average of 20 perpendicular lengths measured per left and right ventricle (RV) using Fiji software v1.53 (National Institutes of Health, Bethesda, MD, USA). The values were normalized for fetal weight (Gussenhoven et al., 2019). The microscopic analysis was performed in six images at 40× magnification (Objective N‐Achroplan 40x/0.25 M27, 420,940–9901, Carl Zeiss, Oberkochen, Germany); all nuclei were counted per image, and the average per LV and RV per animal was calculated.

##### Immunohistochemistry and analysis

###### Laminin Staining (microvasculature marker)

For cardiac tissue, an adapted protocol from Castillo‐Melendez was used to examine micro‐capillarization in the myocardia (Castillo‐Melendez et al., 2017). The same protocol as described for brain tissue laminin staining was applied. Laminin‐positive areas were measured in both the RV and left ventricle (LV) in six images at 10× magnification using Fiji software v1.53 (National Institutes of Health).

###### Ki67 staining (cell proliferation marker)

For cardiomyocyte proliferation, heart sections were deparaffinized using xylene and rehydrated with decreasing concentrations of ethanol. The sections were then rinsed in PBS and undergo antigen retrieval by incubation in 10 mM TRIS/1 mM EDTA buffer (pH 9) at 95°C for 15 min, followed by cooling to room temperature for 30 min. Endogenous peroxidase activity was blocked by incubating the sections in 0.3% H_2_O_2_ in PBS at room temperature for 5 min. After washing in PBS, sections were incubated for 60 min at room temperature with rabbit anti‐Ki67 antibody (1:50; RM‐9106, Thermo Fisher Scientific, Waltham, MA, USA) in PBS/1% BSA. The sections were rewashed with PBS and incubated with a secondary goat anti‐rabbit HRP antibody (1:100; P044801‐2, Agilent Technologies, Santa Clara, CA, USA) in PBS with 1% BSA and 1% rat serum for 30 min. After another washing step in PBS, the sections were incubated with a tertiary rabbit anti‐goat antibody (1:100; P044901‐2, Agilent Technologies, Santa Clara, CA, USA) in PBS with 1% BSA and 1% rat serum, followed again by a wash step in PBS. The specific staining was visualized using DAB (J62216, Thermo Fisher Scientific, Waltham, MA, USA), and nuclei were counterstained with hematoxylin for 10 s before mounting with DEPEX (18,243, Serva Electrophoresis GmbH, Heidelberg, Germany) and cover glass.

The total number of cardiomyocytes with Ki67 staining was counted in six fields per ventricle at 10× magnification using Fiji software v1.53 by two researchers. The average per field was compared between the investigators and re‐analyzed when the difference was larger than 10%.

#### 2.7.3 Kidney

##### Histological techniques and analysis

The left kidneys were sectioned in the longitudinal direction at 5 μm thickness for H&E staining. Full‐section images were taken using a NanoZoomer S60 Digital slide scanner (Hamamatsu Photonics, Hamamatsu city, Japan). All analyses to measure the stage of glomerular maturation, glomerular area, and glomerular density were performed in QuPath 0.5.0. We assessed the glomerular maturation using a human‐based scoring system (by the lack of one in rats) (Sutherland et al., 2011); (Supplemental data method section). This scoring system comprises three stages (Figure S1): the immature comma/S‐shaped stage (S; defined by a variety of different cell types clustered together), the immature capillary loop stage (C; recognized by a row of developing podocytes lined up in a string‐like form), and the mature stage (M; identified by podocytes distributed evenly throughout the glomerulus). We determined the glomerular area in all glomeruli per kidney and analyzed them per maturation stage (Papazova et al., 2018). Glomerular density was determined as the total number of glomeruli per total area per kidney adaptation of (De Winter et al., 2020). The early developmental state of the kidney made it impossible to distinguish yet between cortex or medulla or to determine the nephrogenic zone.

##### Immunohistochemistry and analysis

###### JG12 Staining (microvascular marker)

For kidney sections, immunohistochemistry was performed to examine the microvascular status using JG12 antibodies directed against endothelial aminopeptidase. Sections wete deparaffinized using xylene and rehydrated in decreasing ethanol concentrations. Endogenous peroxidase activity was blocked by incubating the sections in 0.3% H_2_O_2_ in PBS at room temperature for 20 min, followed by rinsing in PBS. Antigen retrieval was done by incubating the sections in 10 mM citrate buffer (pH 6) at 95°C for 20 min, followed by cooling on ice for 30 min. The sections were then rinsed in 0.05% Tween‐20 in PBS and blocked with SuperBlock™ blocking buffer (37,580, Thermo Fisher Scientific, Waltham, MA, USA). Sections were incubated for 60 min at room temperature with mouse anti‐JG12 antibody (1:400; BMS1104, Bender Medsystems) diluted in PBS/1%BSA. After washing in PBS/Tween, sections were incubated with a secondary goat anti‐mouse Brightvision‐HRP antibody (ready‐to‐use solution; DPVM55HRP, ImmunoLogic, Arnhem, the Netherlands) at room temperature for 30 min. The antibody‐specific staining was visualized using Vector NovaRED® Substrate Kit (SK‐4800, Vector Laboratories, Newark, CA, USA), and sections were counterstained with hematoxylin for 10 s before mounting with DEPEX (18,243, Serva Electrophoresis GmbH, Heidelberg, Germany) and cover glass.

###### Ki67 Staining (cell proliferation marker)

For Ki67 staining in kidney sections, a similar deparaffinization and rehydration process was followed. After blocking endogenous peroxidase activity and performing antigen retrieval as described for JG12 staining, sections were incubated with rabbit anti‐Ki67 antibody (1:200; RM‐9106, Thermo Fisher Scientific, Waltham, MA, USA) in PBS/1%BSA for 60 min at room temperature. Following washing in PBS/Tween, sections were incubated with HRP‐conjugated goat anti‐rabbit antibody (1:100; P0448, Agilent Technologies, Santa Clara, CA, USA) for 30 min at room temperature. Staining was enhanced and visualized using NovaRED® Substrate Kit (SK‐4800, Vector Laboratories, Newark, CA, USA), and sections were counterstained with hematoxylin for 10 s before mounting with DEPEX (18,243, Serva Electrophoresis GmbH, Heidelberg, Germany) and cover glass. Full‐section images were taken using a NanoZoomer S60 Digital slide scanner (Hamamatsu, Hamamatsu City, Japan).

Analyses of renal tissue were performed in QuPath 0.5.0 to determine the percentage of positively stained surface area for Ki67 or JG12 for the whole kidney. The total number of Ki67‐ and JG12‐stained pixels was measured using a manually‐set threshold to detect positive staining per selected kidney of interest. The percentage of positively stained area per total kidney area was calculated and presented as fold change.

### 2.8 Statistical analysis

Data are presented as mean ± standard deviation (SD). All statistical analyses were performed using GraphPad Prism v10.0.2 for Windows (GraphPad Software, Boston, MA, USA). Normal distribution was checked using the Shapiro–Wilk normality test. Two‐tailed Unpaired Student's *t*‐tests or, in event of unequal variances, two‐tailed Mann–Whitney tests were used for comparison of two groups. Two‐sided *p*‐values <0.05 were considered statistically significant. For sex differences, a two‐way ANOVA was used with post‐hoc Sidak's multiple comparisons test if applicable.

There was not one specific primary outcome as we performed a multi‐organ approach on which we could base our a priori power calculation. A post‐hoc analysis of fetal weight with a two‐tailed alpha of 0.05 showed a power of 0.92.

## 3 RESULTS

### 3.1 The RUPP procedure causes FGR in the E19 fetuses

Firstly, we aimed to validate the phenotype of FGR and maternal preeclampsia in our model of RUPP surgery. Indeed, we found that the RUPP procedure was represented by a decrease in fetal body weight, fetal crown‐rump length, and placental weight at E19 compared to fetuses of sham‐operated dams (Table 1). In addition, RUPP dams exhibited hypertension and a higher percentage of fetal resorptions compared to sham dams. Maternal levels of protein in the 24‐h urine samples collected at E17 did not significantly differ between the RUPP and sham groups. These maternal and fetal outcomes indicated a successful placental insufficiency‐based FGR model in the RUPP rats.

**TABLE 1:** Validation of RUPP phenotype measured at E19.

	Sham (*n* = 13)	RUPP (*n* = 16)	*p*‐value
Mean arterial blood pressure (mmHg)	114.4 ± 11.4	124.4 ± 10.5	0.02
Proteinuria (mg/ml)^a^	0.46 ± 0.21	0.61 ± 0.28	0.13
Viable litter size E19 (*n*)	12.0 ± 3.0	7.3 ± 3.6	<0.001
Resorptions E14‐19 (%)^b^	10.6 ± 12.2	39.6 ± 22.8	<0.001
Fetal weight (g)	2.23 ± 0.25	1.93 ± 0.21	0.001
Fetal crown‐rump length (cm)^c^	3.92 ± 0.21	3.43 ± 0.31	<0.0001
Placental weight (g)	0.49 ± 0.09	0.41 ± 0.05	<0.01
Fetal: placental weight ratio	4.64 ± 0.73	4.64 ± 0.44	0.98

*Note*: Data are shown as mean ± SD. E, embryonic day; RUPP, reduced uterine perfusion pressure.

^a^ *n* = 12 sham and *n* = 15 RUPP were used due to a problem with sampling.

^b^ *n* = 12 sham were used due missing the amount of viable pups at E14 of one sham dam.

^c^ *n* = 15 RUPP were used due to missing one fetal length of one fetus.

### 3.2 Histology: Detecting multi‐organ structural alterations in RUPP fetuses

Secondly, we aimed to assess potential early structural and vascular changes in the brain, heart, and kidney of RUPP fetuses using a multi‐organ approach as described in the subsequent paragraphs. No sex‐specific differences in any of the outcomes were observed (Tables S3‐S5).

#### 3.2.1 Brain: RUPP Decreases fetal brain area and cortical thickness

**FIGURE 1:**
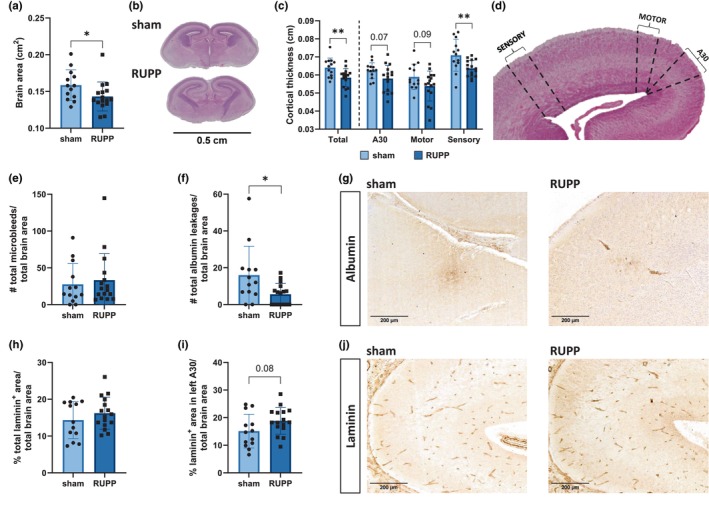
RUPP fetuses show changes in morphological brain structure at E19. (a) RUPP fetuses displayed decreased brain area compared to sham fetuses. (b) A macroscopic example of the cerebral area between sham and RUPP using H&E staining. (c) RUPP fetuses showed a decreased total cortical thickness, especially in the somatosensory cortex. (d) Scheme of regions where cortical thickness was measured. (e) No changes in the total number of microbleeds in the total brain area were observed between sham and RUPP groups (typical histological examples of cerebral microbleeds are shown in Figure S2. (f) Sham fetuses exhibited a higher number of albumin leakages compared to the RUPP group. (g) Microscopic examples of albumin extravasations in both sham and RUPP groups. (h, i) No difference in the total laminin‐positive area in the total brain area was observed. (j) Microscopic examples of laminin coverage in the A30 region of the left hemisphere. Data (*n* = 13 sham vs. *n* = 16 RUPP) are shown as mean ± SD. * and ** represent a *p*‐value of <0.05 and <0.01 respectively. E19, embryonic day 19; H&E, hematoxylin and eosin; RUPP, reduced uterine perfusion pressure; #, number.

At E19, RUPP fetuses revealed a significant decrease in brain area (Figure 1a,b) and decreased total cortical thickness (Figure 1c,d). Out of the three cortical regions assessed, the decrease in cortical thickness was mainly observed in the sensory cortex. The total number of cerebral microbleeds was similar between the two groups (Figure 1e; typical examples shown in Figure S2). In order to investigate whether the RUPP procedure led to an increased BBB permeability, the amount of albumin leakages were quantified. The sham fetuses displayed a higher amount of albumin leakages in the brain compared to the RUPP group (Figure 1f,g). Lastly, no difference in the percentage of laminin coverage, as a measure of cerebral vascularization, between sham and RUPP groups was observed (Figure 1h–j). The data in Figure 1 indicated that RUPP E19 fetuses presented structural differences, but no obvious vascularization problems, revealed by RUPP fetuses displaying a smaller brain area, thinner sensory cortex, decreased number of albumin leakages, and no difference in laminin coverage compared to sham.

#### 3.2.2 Heart: Subtle changes in cardiac morphology after RUPP but no changes in proliferation and vascularization

**FIGURE 2:**
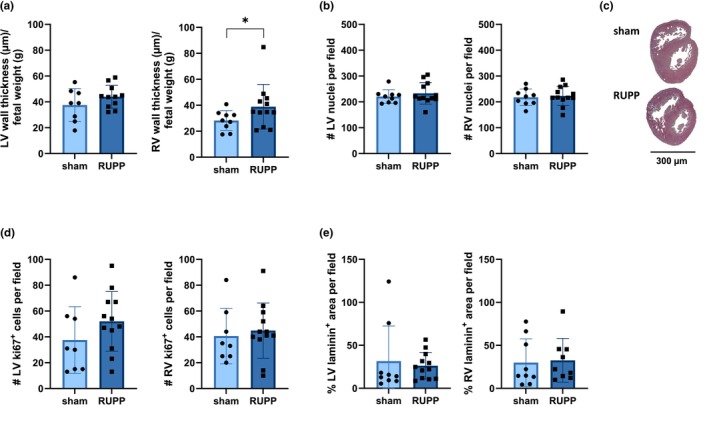
Cardiac analysis shows macroscopic morphology changes in RUPP fetuses at E19. (a) The wall thickness is larger in RUPP fetuses compared to sham in the right but not left ventricle. (b) No difference between groups in the number of cardiomyocytes in LV and RV. (c) A macroscopic example of H&E staining to indicate the difference in ventricular wall thickness. (d) No difference in cell proliferation as the number of Ki67‐positive cells appeared to be similar in RUPP and sham fetuses. (e) No differences in laminin coverage were observed between sham and RUPP fetuses. Typical histological examples of H&E, Ki67 and laminin stainings are shown in Figure S3. Data (*n* = 8 sham, *n* = 12 RUPP) are shown as mean ± SD. * represents *p* < 0.05. E19, embryonic day 19; g, gram; H&E, hematoxylin and eosin; LV, left ventricle; RUPP, reduced uterine perfusion pressure; RV, right ventricle; #, number.

The cardiac morphology was evaluated. Therefore, first, the LV and RV wall thicknesses were determined. Interestingly, the RV wall thickness was significantly increased in RUPP fetuses, indicating some form of hypertrophic response (Figure 2a,c). Next, to assess the level of proliferation in cardiac tissue, the number of nuclei per field in H&E staining and the number of Ki67‐positive cells per field were counted. For both stainings, no differences in either LV or RV between sham and RUPP were observed, suggesting no overall changes in cell proliferation (Figure 2b,d). With regards to vascularization, a laminin staining was used. No significant differences in levels of vascularization in both LV and RV between the two experimental groups were detected (Figure 2e). Microscopic examples of H&E, Ki67 and laminin stainings are displayed in supplemental material (Figure S3). In summary, these findings suggest that there are significant structural changes in the RV but no differences at microscopic level in vascularization and cell profileration.

#### 3.2.3 Kidney: Similar glomerular morphology, proliferation, and glomerular capillary density after RUPP

**FIGURE 3:**
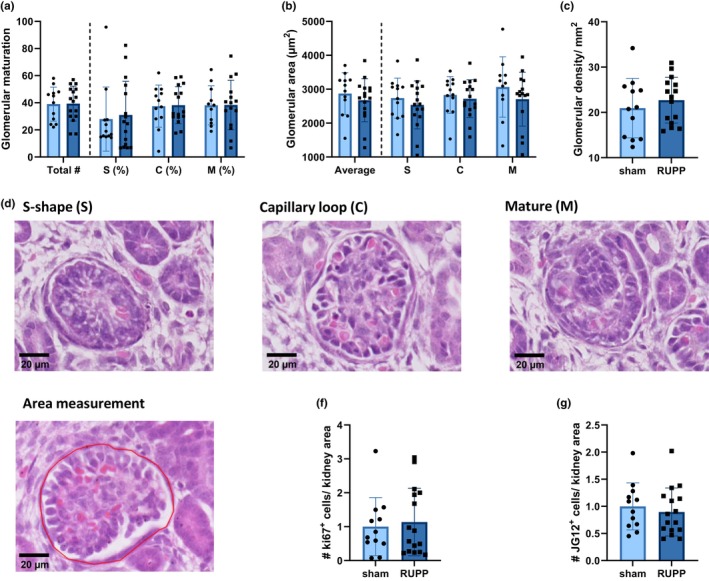
No differences in vascular or structural development in the kidneys between RUPP and sham fetuses at E19. (a) No differences regarding the glomerular stage of maturation, total number of glomeruli and percentage of each stage, assessed with H&E, between sham and RUPP fetuses were observed. (b) The glomerular area in total and per individual stage of maturation were similar between the sham and RUPP groups. (c) No difference in the glomerular density was observed. (d) Microscopic examples of the different stages of glomerular maturation. (e) Microscopic example of how glomerular area was measured. (f) The cellular proliferation rate as measured with Ki67 was similar between the groups. (g) The glomerular capillary density measured as JG12‐positive cells did not show any difference between the sham and RUPP groups. Typical histological examples of Ki67 and JG12 stainings are shown in Figure S4. Data (*n* = 12 sham vs. *n* = 16 RUPP) are shown as mean ± SD. C, capillary loop; E19, embryonic day 19; H&E, hematoxylin and eosin; M, mature; RUPP, reduced uterine perfusion pressure; S, s‐shape; #, number.

Lastly, glomerular morphology, proliferation, and glomerular capillary density through different histological stainings were assessed in the fetal kidneys. With an H&E staining, glomerular maturation, glomerular area and glomerular density were measured. No significant differences in any of the three outcome parameters between sham and RUPP fetuses at E19 were observed (Table S6; Figure 3a–e). Next, Ki67 staining as a measure of cell proliferation in the kidney tissue and JG12 staining to assess glomerular capillary density in renal tissue were performed. No significant changes in the total number of Ki67‐positive cells or JG12‐positive cells per kidney area between the two groups were detected, indicating similar levels of cellular proliferation and glomerular capillary density, respectively, in renal tissue (Figure 3f,g). Typical examples of Ki67 and JG12 stainings are shown in supplemental material Figure(S4). Taken together, these findings suggest minor to negligible differences in renal development in RUPP fetuses.

## 4 DISCUSSION

This study in growth‐restricted fetuses derived from a placental insufficiency rat model shows no evident vascular differences between RUPP and sham fetuses at E19. We did reveal several subtle structural differences in the brain (decreased brain area, cortical thickness, and albumin leakages in RUPP) and heart (increased right ventricular wall thickness), but not in the kidneys. Our multi‐organ approach therefore suggests that the fetal brain reacts more sensitively to placental insufficiency after the RUPP surgery compared to the heart and kidneys, while maintaining a protective or compensatory vascular mechanism.

### 4.1 The lack of differences in fetal organ vascularization: A compensatory mechanism or too early to detect subclinical adapations?

Since our study did not reveal vascular changes in any of the organs, this could suggest that RUPP fetuses possess a compensatory mechanisms to protect against chronic hypoxia and circulatory fluctuations. For instance, our observed increase in ventricular wall thickness might be a first sign of cardiac remodeling, but this phenomenon has also been considered a temporary adaptation to overcome the higher placental restistance in placental insufficiency syndromes (Freedman et al., 2023; Murotsuki et al., 1997; Pérez‐Cruz et al., 2015; Youssef et al., 2020) that potentially normalizes at birth (Kiserud et al., 2006; Meent et al., 2024). The same amount of cardiomyocyte nuclei and similar microvascular status of the heart between RUPP and sham supports a fully compenssatory adaptation in redistribution of fetal circulation toward the heart. Our data are consistent with one study showing that FGR induces only vascular remodeling of the fetal arteries of the lower extremities/body with sparing of the fetal arteries in the upper body (Cañas et al., 2017), which might also explain the sparing of the kidneys. Unfortunately we were unable to compare our results on vascularisation in the fetal heart and kidneys due to the lack of studies specifically addressing the heart and kidney. The well‐known brain‐sparing response might also have maintained the structrual integrity of the BBB, especially since previous studies consider laminin a good indicator of the overall BBB integrity, and we observed no difference in laminin density (Zapata‐Acevedo et al., 2022).

In contrast to our results, numerous studies reveal a disordered NVU and an increased BBB permeability in FGR (Wu et al., 2024a). However, these studies are performed in larger animal models of FGR (piglet and lamb) and use low percentages of isoflurane anesthesia (Castillo‐Melendez et al., 2015; Castillo‐Melendez et al., 2017; Chand et al., 2022; Malhotra et al., 2018; Malhotra et al., 2020; Wu et al., 2024b; Yawno et al., 2019), whereas the RUPP surgery is performed under relatively higher percentage of isoflurane at least during the induction period. Given that isoflurane exhibits neuroprotective properties in the presence of a predisposition to brain injury (Burchell et al., 2013), this might have prevented brain impairment in the RUPP group. Nevertheless, the one study performed in RUPP fetuses that studied the BBB permeability with cerebral water content, did not demonstrate an increased BBB permeability in the RUPP, which is in line with our study (Giambrone et al., 2019). The same study showed an increase in the number of microbleeds in the posterior part of the fetal RUPP brain only, and not in the anterior part of the brain as we investigated in our study (Giambrone et al., 2019).

The lack of vascular differences in our study might also be due to the fetal timing, as 5 days after the induction of placental insufficiency might be too early to detect vascular adaptations in any of the three organs of the FGR fetuses. This might also explain the lack of differences in the kidneys, especially considering that the kidneys are already borderline hypoxic under normal circumstances in combination with posseing a major renal reserve before function changes (Gaipov et al., 2016; Ronco et al., 2017; Sharma et al., 2014). In this study, the reduced uteroplacental perfusion exposes the RUPP fetuses for the last 5 days, while in humans this negative environment is present from the start. The studies that demonstrated an affected NVU were performed in larger animal models of FGR with relatively longer exposure to placental hypoxia, which also supports this concept (Castillo‐Melendez et al., 2015; Castillo‐Melendez et al., 2017; Chand et al., 2022; Malhotra et al., 2018; Malhotra et al., 2020; Yawno et al., 2019).

Nevertheless, the exposure to hypoxia, decreased nutrition supply and temporary altered perfusion might still result in epigenetic changes during these 5 days, as we did oberve some structural changes (which are in line with previous studies (Delcour et al., 2012; Dubois et al., 2008; Miller et al., 2016; Tolsa et al., 2004)) already visible after 5 days of exposure to placental insufficiency in RUPP fetuses at E19. Epigenetic differences in organ microvascularisation might increase the susceptibility of developing non‐communicable diseases by epigenetic pathways in adulthood. A second hit, such as an unhealthy diet with high protein or cholesterol, obesity, illness or (nephro)toxic medication across the lifespan might eventually lead to cardio‐renal or neurovascular diseases in FGR offspring (Ransom et al., 2024). Organ microvasculature and arterial stiffness might mediate this process (Sehgal, 2011).

Besides epigenetics, other overlapping mechanisms could be at play in placental insufficiency‐induced FGR. Interest has grown in inflammatory pathways as common mechanisms since inflammatory and angiogenesis closely interact in the pathogenesis of placental dysfunction (Collier et al., 2021; Rana et al., 2022). While beyond the scope of our study, studies suggest neuroinflammation as the key mechanism of NVU disruption and altered vascularity in correlation with long‐term sequela in FGR offspring (Wu et al., 2024a). Although not studied in FGR, interstitial inflammation has been shown to affect the renal microvascularisation in salt‐depending hypertension (Rodriguez‐Iturbe & Johnson, 2010) and cytokines modulate microvascular coronary function (Dal Lin et al., 2015).

### 4.2 Strength and limitations

The strength of our study includes the vascular overlap in a multi‐organ approach instead of investigating the response to placental hypoxia in only one organ. We also used the RUPP rat model, a well‐established animal model of placental insufficiency‐induced FGR, which we validated for both the maternal and fetal phenotype. To our knowledge, we are the first to study the capillary density in the brain and kidney in the RUPP model. And, as for the heart, we are the first to study capillary density in FGR models in general. In addition, our adapted scoring system for glomerular maturation is unique as it is specifically designed for rats, which has not been previously done. We maximized the reproducibility and transparency by following the ARRIVE guidelines.

We also acknowledge some limitations. While FGR in the human condition knows an arbitrary division in early or late onset, the induction of the RUPP model at E14 corresponds to mid‐gestation. While beyond the scope of our study, we could not correlate the histological differences to long‐term organ function. We selected the brain and heart as organs that receive more perfusion as a consequence of fetal circulatory redistribution. Kidneys were also selected as they are one of the lesser perfused organs, are well studied in FGR, and relatively easy to collect; nevertheless, other organs could have fit the latter category as well. We focused on histological measurements in different organs and therefore not on gene or protein expression alterations in molecular pathways.

### 4.3 Perspectives and significance

The RUPP model is a suitable model to study multi‐organ development in placental insufficiency‐induced FGR. Potentially the RUPP fetuses execute compensatory mechanisms wherefore no robust microvascular adaptations at organ level were detectable at this fetal stage yet. This does not exclude that potential subclinical adaptations might progress to clinical manifestations after exposure to a second hit later in life. Our study also reveals that FGR mostly impacts structural alterations in the fetal brain and leads to structural adapations in the heart, with sparing of renal development. These changes in fetal organ development might be the consequence of subclinical vascular deterioration as a response to chronic hypoxia and circulatory redistribution; alternatively, other mechanisms, such as inflammatory pathways or epigenetics, might be at play. With the higher likelihood of an increased susceptibility rather than a detectable presence of detoriation at this fetal stage, this research highlights the need for future studies in the RUPP model as well as in large human cohort studies to examine the impact of the second‐hits. Future research toward unraveling the underlying mechanisms should include a multi‐organ approach to develop therapeutic agents to prevent lifelong burden of multiple organ systems. Since organ microvasculature and arterial stiffness might mediate this process, it could be of interest as novel therapeutic targets (Sehgal, 2011). Another promising novel therapeutic strategy might involve anti‐inflammatory therapies since studies suggest neuroinflammation as a key mechanism of NVU disruption and altered vascularity in correlation with long‐term sequelae in FGR offspring (Wu et al., 2024a). A promising three‐dimensional imaging technique includes micro‐CT, which visualizes vascularization of multiple fetal organs at once in vivo (Holdsworth & Thornton, 2002). In addition, the longitudinal follow‐up of vascular organ development in combination with organ function aids in pinpointing the optimal timing for preventive strategies.

## AUTHOR CONTRIBUTIONS

Conceived and designed research (J.A.R, C.M.K, A.T.L, C.H.A.N, F.T), performed experiments (J.A.R, C.M.K, K.N, D.V, D.S, K.A, F.T), analyzed data (J.A.R, C.M.K, D.S, K.N, F.T), interpreted results of experiments (J.A.R, C.M.K, K.N, M.K, A.T.L, F.E.H, C.H.A.N, F.T), prepared figures (J.A.R, C.M.K, K.N), drafted manuscript (J.A.R, FT), edited and revised manuscript (J.A.R, C.M.K, K.N, D.V, K.A, D.S, M.K, A.T.L, C.H.A.N, F.E.H, F.T), approved final version of manuscript (J.A.R, C.M.K, K.N, D.V, K.A, D.S, M.K, A.T.L, F.E.H, C.H.A.N, F.T).

## FUNDING INFORMATION

This project has received funding from the Wilhelmina Children's Hospital, department of Pediatrics, Booster grant (to ATL), Wilhelmina Children's Hospital, department of Pediatrics, Clinical Research Fellowship 2023 (to FT), Dutch Kidney Foundation, Student internship grant: 23OSR026 (to KN), European Union's Horizon 2020 Research and Innovation programme PREMSTEM under Grant Agreement No 874721 (to CHAN), and ZonMW MKMD neutral results grant: 114024183 (to FT).

## CONFLICT OF INTEREST STATEMENT

The authors declare no conflict of interest, financial or otherwise.

## Supporting information


Data S1.
Table S1.
Table S2.
Figure S1.
Table S3.
Table S4.
Table S5.
Figure S2.
Figure S3.
Figure S4.
Table S6.

## Data Availability

All data are provided within the manuscript or supplemental material.

## References

[phy270244-bib-0001] Alexander, B. T. (2003). Placental insufficiency leads to development of hypertension in growth‐restricted offspring. Hypertension, 41, 457–462. 10.1161/01.HYP.0000053448.95913.3D 12623943

[phy270244-bib-0002] Barker, D. J. P. (2006). Adult consequences of fetal growth restriction. Clinical Obstetrics and Gynecology, 49, 270–283. 10.1097/00003081-200606000-00009 16721106

[phy270244-bib-0003] Burchell, S. R. , Dixon, B. J. , Tang, J. , & Zhang, J. H. (2013). Isoflurane provides neuroprotection in neonatal hypoxic ischemic brain injury. Journal of Investigative Medicine, 61, 1078–1083. 10.2310/JIM.0B013E3182A07921 23884213 PMC3785571

[phy270244-bib-0004] Calkins, K. , & Devaskar, S. U. (2011). Fetal origins of adult disease. Current Problems in Pediatric and Adolescent Health Care, 41, 158–176. 10.1016/j.cppeds.2011.01.001 21684471 PMC4608552

[phy270244-bib-0005] Cañas, D. , Herrera, E. A. , García‐Herrera, C. , Celentano, D. , & Krause, B. J. (2017). Fetal growth restriction induces heterogeneous effects on vascular biomechanical and functional properties in Guinea pigs (Cavia porcellus). Frontiers in Physiology, 8, 144. 10.3389/fphys.2017.00144 28344561 PMC5344887

[phy270244-bib-0006] Castillo‐Melendez, M. , Yawno, T. , Allison, B. J. , Jenkin, G. , Wallace, E. M. , & Miller, S. L. (2015). Cerebrovascular adaptations to chronic hypoxia in the growth restricted lamb. International Journal of Developmental Neuroscience, 45, 55–65. 10.1016/j.ijdevneu.2015.01.004 25639519

[phy270244-bib-0007] Castillo‐Melendez, M. , Yawno, T. , Sutherland, A. , Jenkin, G. , Wallace, E. M. , & Miller, S. L. (2017). Effects of antenatal melatonin treatment on the cerebral vasculature in an ovine model of fetal growth restriction. Developmental Neuroscience, 39, 323–337. 10.1159/000471797 28467985

[phy270244-bib-0008] Chand, K. K. , Miller, S. M. , Cowin, G. J. , Mohanty, L. , Pienaar, J. , Colditz, P. B. , Bjorkman, S. T. , & Wixey, J. A. (2022). Neurovascular unit alterations in the growth‐restricted newborn are improved following ibuprofen treatment. Molecular Neurobiology, 59, 1018–1040. 10.1007/s12035-021-02654-w 34825315

[phy270244-bib-0009] Collier, A. r. Y. , Smith, L. A. , & Karumanchi, S. A. (2021). Review of the immune mechanisms of preeclampsia and the potential of immune modulating therapy. Human Immunology, 82, 362–370. 10.1016/J.HUMIMM.2021.01.004 33551128 PMC8062309

[phy270244-bib-0010] Crepel, V. , Represa, A. , & Ben‐Ari, Y. (1988). Effect of ischemia and intra‐amygdaloid kainate injection on the density of NMDA binding sites in the hippocampal CA1 region. European Journal of Pharmacology, 151, 355–356. 10.1016/0014-2999(88)90827-8 2844561

[phy270244-bib-0011] Dal Lin, C. , Tona, F. , & Osto, E. (2015 2015.). Coronary microvascular function and beyond: The crosstalk between hormones, cytokines, and neurotransmitters. International Journal of Endocrinology, 2015, 1–17. 10.1155/2015/312848 PMC4466475 26124827

[phy270244-bib-0012] De Winter, D. , Salaets, T. , Gie, A. , Deprest, J. , Levtchenko, E. , & Toelen, J. (2020). Glomerular developmental delay and proteinuria in the preterm neonatal rabbit. PLoS One, 15, e0241384. 10.1371/journal.pone.0241384 33166318 PMC7652305

[phy270244-bib-0013] Delcour, M. , Olivier, P. , Chambon, C. , Pansiot, J. , Russier, M. , Liberge, M. , Xin, D. , Gestreau, C. , Alescio‐Lautier, B. , Gressens, P. , Verney, C. , Barbe, M. F. , Baud, O. , & Coq, J. O. (2012). Neuroanatomical, sensorimotor and cognitive deficits in adult rats with white matter injury following prenatal ischemia. Brain Pathology, 22, 1–16. 10.1111/j.1750-3639.2011.00504.x 21615591 PMC8028965

[phy270244-bib-0014] Dhakal, P. , & Soares, M. J. (2017). Single‐step PCR‐based genetic sex determination of rat tissues and cells. BioTechniques, 62, 232–233. 10.2144/000114548 28528577 PMC5831150

[phy270244-bib-0015] Dubois, J. , Benders, M. , Cachia, A. , Lazeyras, F. , Ha‐Vinh Leuchter, R. , Sizonenko, S. V. , Borradori‐Tolsa, C. , Mangin, J. F. , & Hüppi, P. S. (2008). Mapping the early cortical folding process in the preterm newborn brain. Cerebral Cortex, 18, 1444–1454. 10.1093/cercor/bhm180 17934189

[phy270244-bib-0016] Figueras, F. , Cruz‐Martinez, R. , Sanz‐Cortes, M. , Arranz, A. , Illa, M. , Botet, F. , Costas‐Moragas, C. , & Gratacos, E. (2011). Neurobehavioral outcomes in preterm, growth‐restricted infants with and without prenatal advanced signs of brain‐sparing. Ultrasound in Obstetrics and Gynecology, 38, 288–294. 10.1002/uog.9041 21557369

[phy270244-bib-0017] Freedman, A. A. , Price, E. , Franklin, A. , & Ernst, L. M. (2023). Measures of fetal growth and cardiac structure in stillbirths with placental maternal vascular Malperfusion: Evidence for heart weight sparing and structural cardiac alterations in humans. Pediatric and Developmental Pathology, 26, 310–317. 10.1177/10935266231166548 37082927

[phy270244-bib-0018] Gaipov, A. , Solak, Y. , Zhampeissov, N. , Dzholdasbekova, A. , Popova, N. , Molnar, M. Z. , Tuganbekova, S. , & Iskandirova, E. (2016). Renal functional reserve and renal hemodynamics in hypertensive patients. Renal Failure, 38, 1391–1397. 10.1080/0886022X.2016.1214052 27470640

[phy270244-bib-0019] Giambrone, A. B. , Logue, O. C. , Shao, Q. , Bidwell, G. L. , & Warrington, J. P. (2019). Perinatal micro‐bleeds and neuroinflammation in e19 rat fetuses exposed to utero‐placental ischemia. International Journal of Molecular Sciences, 20(16), 4051. 10.3390/ijms20164051 31434191 PMC6720786

[phy270244-bib-0020] Giussani, D. A. (2016). The fetal brain sparing response to hypoxia: Physiological mechanisms. The Journal of Physiology, 594, 1215–1230. 10.1113/JP271099 26496004 PMC4721497

[phy270244-bib-0021] Granger, J. P. , LaMarca, B. B. D. , Cockrell, K. , Sedeek, M. , Balzi, C. , Chandler, D. , & Bennett, W. (2006). Reduced uterine perfusion pressure (RUPP) model for studying cardiovascular‐renal dysfunction in response to placental ischemia. Methods in Molecular Medicine, 122, 383–392. 10.1385/1-59259-989-3:381 16511995

[phy270244-bib-0022] Gussenhoven, R. , Klein, L. , Ophelders, D. R. M. G. , Habets, D. H. J. , Giebel, B. , Kramer, B. W. , Schurgers, L. J. , Reutelingsperger, C. P. M. , & Wolfs, T. G. A. M. (2019). Annexin A1 as neuroprotective determinant for blood‐brain barrier integrity in neonatal hypoxic‐ischemic encephalopathy. Journal of Clinical Medicine, 8(2), 137. 10.3390/jcm8020137 30682787 PMC6406389

[phy270244-bib-0023] Herrera, E. A. , & González‐Candia, A. (2021). Gestational hypoxia and blood‐brain barrier permeability: Early origins of cerebrovascular dysfunction induced by epigenetic mechanisms. Frontiers in Physiology, 12, 717550. 10.3389/FPHYS.2021.717550 34489733 PMC8418233

[phy270244-bib-0024] Holdsworth, D. W. , & Thornton, M. M. (2002). Micro‐CT in small animal and specimen imaging. Trends in Biotechnology, 20, S34–S39. 10.1016/S0167-7799(02)02004-8

[phy270244-bib-0025] Kammen, C. M. , Taal, S. E. L. , Wever, K. E. , Granger, J. P. , Lely, A. T. , Terstappen, F. , & Kammen, C. M. (2024). The reduced uterine perfusion pressure as a model for preeclampsia and fetal growth restriction in murine: A systematic review and meta‐analysis. American journal of Physiology Heart Circulation, 327(1), 89–107. 10.1152/ajpheart.00056.2024 PMC11380978 38758122

[phy270244-bib-0026] Kiserud, T. , Ebbing, C. , Kessler, J. , & Rasmussen, S. (2006). Fetal cardiac output, distribution to the placenta and impact of placental compromise. Ultrasound in Obstetrics & Gynecology, 28, 126–136. 10.1002/UOG.2832 16826560

[phy270244-bib-0027] Li, J. , Lamarca, B. , & Reckelhoff, J. F. (2012). A model of preeclampsia in rats: The reduced uterine perfusion pressure (RUPP) model. American Journal of Physiology. Heart and Circulatory Physiology, 303(1), H1–H8. 10.1152/ajpheart.00117.2012 ‐Preeclampsia. 22523250 PMC3404644

[phy270244-bib-0028] Malhotra, A. , Allison, B. J. , Castillo‐Melendez, M. , Jenkin, G. , Polglase, G. R. , & Miller, S. L. (2019). Neonatal morbidities of fetal growth restriction: Pathophysiology and impact. Front Endocrinol (Lausanne), 10, 55. 10.3389/FENDO.2019.00055 30792696 PMC6374308

[phy270244-bib-0029] Malhotra, A. , Castillo‐Melendez, M. , Allison, B. J. , Sutherland, A. E. , Nitsos, I. , Pham, Y. , Alves De Alencar Rocha, A. K. , Fahey, M. C. , Polglase, G. R. , Jenkin, G. , Miller, S. L. , Castillo‐Melendez, M. A. , Bj, A. , Ae, S. , Pham, N. I. , Alencar, A. , Ak, R. , & Mc, F. (2018). Neuropathology as a consequence of neonatal ventilation in premature growth‐restricted lambs. American Journal of Physiology. Regulatory, Integrative and Comparative Physiology, 315, 1183–1194. 10.1152/ajpregu.00171 30230932

[phy270244-bib-0030] Malhotra, A. , Castillo‐Melendez, M. , Allison, B. J. , Sutherland, A. E. , Nitsos, I. , Pham, Y. , McDonald, C. A. , Fahey, M. C. , Polglase, G. R. , Jenkin, G. , & Miller, S. L. (2020). Neurovascular effects of umbilical cord blood‐derived stem cells in growth‐restricted newborn lambs: UCBCs for perinatal brain injury. Stem Cell Research & Therapy, 11(1), 17. 10.1186/s13287-019-1526-0 31915068 PMC6947982

[phy270244-bib-0031] Meent, M. , Nijholt, K. T. , Joemmanbaks, S. C. A. , Kooiman, J. , Schipper, H. S. , Wever, K. E. , Lely, A. T. , & Terstappen, F. (2024). Understanding changes in echocardiographic parameters at different ages following fetal growth restriction: A systematic review and meta‐analysis. American Journal of Physiology. Heart and Circulatory Physiology, 326, 1469–1488. 10.1152/AJPHEART.00052.2024 PMC11380958 38668703

[phy270244-bib-0032] Miller, S. L. , Huppi, P. S. , & Mallard, C. (2016). The consequences of fetal growth restriction on brain structure and neurodevelopmental outcome. The Journal of Physiology, 594, 807–823. 10.1113/JP271402 26607046 PMC4753264

[phy270244-bib-0033] Murotsuki, J. , Challis, J. R. G. , Han, V. K. M. , Fraher, L. J. , & Gagnon, R. (1997). Chronic fetal placental embolization and hypoxemia cause hypertension and myocardial hypertrophy in fetal sheep. The American Journal of Physiology, 272(1 Pt 2), R201–R207. 10.1152/AJPREGU.1997.272.1.R201 9039010

[phy270244-bib-0034] Nardozza, L. M. M. , Caetano, A. C. R. , Zamarian, A. C. P. , Mazzola, J. B. , Silva, C. P. , Marçal, V. M. G. , Lobo, T. F. , Peixoto, A. B. , & Araujo Júnior, E. (2017). Fetal growth restriction: Current knowledge. Archives of Gynecology and Obstetrics, 295, 1061–1077. 10.1007/S00404-017-4341-9 28285426

[phy270244-bib-0035] Papazova, D. A. , Krebber, M. M. , Oosterhuis, N. R. , Gremmels, H. , Van Zuilen, A. D. , Joles, J. A. , & Verhaar, M. C. (2018). Dissecting recipient from donor contribution in experimental kidney transplantation: Focus on endothelial proliferation and inflammation. Disease Models & Mechanisms, 11(7), dmm035030. 10.1242/dmm.035030 30038062 PMC6078404

[phy270244-bib-0036] Paxinos, G. , & Ashwell, K. (2018). Atlas of the developing rat nervous system. 9780128130582. Elsevier Science.

[phy270244-bib-0037] Pérez‐Cruz, M. , Cruz‐Lemini, M. , Fernández, M. T. , Parra, J. A. , Bartrons, J. , Gõmez‐Roig, M. D. , Crispi, F. , & Gratacõs, E. (2015). Fetal cardiac function in late‐onset intrauterine growth restriction vs small‐for‐gestational age, as defined by estimated fetal weight, cerebroplacental ratio and uterine artery doppler. Ultrasound in Obstetrics and Gynecology, 46, 465–471. 10.1002/uog.14930 26112274

[phy270244-bib-0038] Rana, S. , Burke, S. D. , & Karumanchi, S. A. (2022). Imbalances in circulating angiogenic factors in the pathophysiology of preeclampsia and related disorders. American Journal of Obstetrics and Gynecology, 226, S1019–S1034. 10.1016/J.AJOG.2020.10.022 33096092 PMC8884164

[phy270244-bib-0039] Ransom, K. V. , Traylor, M. K. , Batman, G. B. , Mulekar, M. S. , Hill, B. D. , Nelson, A. R. , & Keller, J. L. (2024). Arterial stiffness mediates the association between age and processing speed at low levels of microvascular function in humans across the adult lifespan. American Journal of Physiology. Heart and Circulatory Physiology, 326, H346–H356. 10.1152/AJPHEART.00662.2023 38038715 PMC11219056

[phy270244-bib-0040] Rock, C. R. , White, T. A. , Piscopo, B. R. , Sutherland, A. E. , Pham, Y. , Camm, E. J. , Sehgal, A. , Polglase, G. R. , Miller, S. L. , & Allison, B. J. (2023). Cardiovascular decline in offspring during the perinatal period in an ovine model of fetal growth restriction. American Journal of Physiology. Heart and Circulatory Physiology, 325, H1266–H1278. 10.1152/AJPHEART.00495.2023 37773057

[phy270244-bib-0041] Rodriguez‐Iturbe, B. , & Johnson, R. J. (2010). The role of renal microvascular disease and interstitial inflammation in salt‐sensitive hypertension. Hypertension Research, 33, 975–980. 10.1038/HR.2010.148 20686485

[phy270244-bib-0042] Ronco, C. , Bellomo, R. , & Kellum, J. (2017). Understanding renal functional reserve. Intensive Care Medicine, 43, 917–920. 10.1007/s00134-017-4691-6 28213622

[phy270244-bib-0043] Sehgal, A. (2011). Haemodynamically unstable preterm infant: An unresolved management conundrum. European Journal of Pediatrics, 170, 1237–1245. 10.1007/S00431-011-1435-4 21424672

[phy270244-bib-0044] Sharma, A. , Mucino, M. J. , & Ronco, C. (2014). Renal functional reserve and renal recovery after acute kidney injury. Nephron Clin Pract, 127, 94–100. 25343829 10.1159/000363721

[phy270244-bib-0045] Tolsa, C. B. , Zimine, S. , Warfield, S. K. , Freschi, M. , Rossignol, A. S. , Lazeyras, F. , Hanquinet, S. , Pfizenmaier, M. , & Hüppi, P. S. (2004). Early alteration of structural and functional brain development in premature infants born with intrauterine growth restriction. Pediatric Research, 56, 132–138. 10.1203/01.PDR.0000128983.54614.7E 15128927

[phy270244-bib-0046] Visentin, S. , Grumolato, F. , Nardelli, G. B. , Di Camillo, B. , Grisan, E. , & Cosmi, E. (2014). Early origins of adult disease: Low birth weight and vascular remodeling. Atherosclerosis, 237, 391–399. 10.1016/J.ATHEROSCLEROSIS.2014.09.027 25463063

[phy270244-bib-0047] Wu, B. A. , Chand, K. K. , Bell, A. , Miller, S. L. , Colditz, P. B. , Malhotra, A. , & Wixey, J. A. (2024a). Effects of fetal growth restriction on the perinatal neurovascular unit and possible treatment targets. Pediatric Research, 95, 59–69. 10.1038/S41390-023-02805-W 37674023 PMC10798895

[phy270244-bib-0048] Yawno, T. , Sutherland, A. E. , Pham, Y. , Castillo‐Melendez, M. , Jenkin, G. , & Miller, S. L. (2019). Fetal growth restriction alters cerebellar development in fetal and neonatal sheep. Frontiers in Physiology, 10, 560. 10.3389/fphys.2019.00560 31191328 PMC6539217

[phy270244-bib-0049] Youssef, L. , Miranda, J. , Paules, C. , Garcia‐Otero, L. , Vellvé, K. , Kalapotharakos, G. , Sepulveda‐Martinez, A. , Crovetto, F. , Gomez, O. , Gratacós, E. , & Crispi, F. (2020). Fetal cardiac remodeling and dysfunction is associated with both preeclampsia and fetal growth restriction. American Journal of Obstetrics and Gynecology, 222, 79. 10.1016/J.AJOG.2019.07.025 31336074

[phy270244-bib-0050] Zapata‐Acevedo, J. F. , García‐Pérez, V. , Cabezas‐Pérez, R. , Losada‐Barragán, M. , Vargas‐Sánchez, K. , & González‐Reyes, R. E. (2022). Laminin as a biomarker of blood‐brain barrier disruption under neuroinflammation: A systematic review. International Journal of Molecular Sciences, 23(12), 6788. 10.3390/IJMS23126788 35743229 PMC9224176

[phy270244-bib-0051] Zohdi, V. , Sutherland, M. R. , Lim, K. , Gubhaju, L. , Zimanyi, M. A. , & Black, M. J. (2012 2012.). Low birth weight due to intrauterine growth restriction and/or preterm birth: Effects on nephron number and long‐term renal health. Int J Nephrol, 2012, 136942. 10.1155/2012/136942 22970368 PMC3434386

