# Supplementary material for: Organ development in growth‐restricted fetuses in the reduced uterine perfusion pressure rat model: A vascular approach of brain, heart, and kidney
Source: Physiol Rep. 2025 Feb 12;13(3):e70244. doi: 10.14814/phy2.70244 (PMC11821727; doi:10.14814/phy2.70244)
Supplement: Supplementary file 1 — Data S1. Table S1. Table S2. Figure S1. Table S3. Table S4. Table S5. Figure S2. Figure S3. Figure S4. Table S6. [file PHY2-13-e70244-s001.docx]

RESEARCH ARTICLE – Supplemental material

running head: vascular and organ development in growth restricted fetuses

Organ development in growth restricted fetuses in the Reduced Uterine Perfusion Pressure rat model: a vascular approach of brain, heart, and kidney

J. Alhama-Riba^1^, C.M. van Kammen^2^, K.T. Nijholt^3^, D. Viveen^1^, K. Amarouchi^1^, D. Shasha^1^, M.M. Krebber^4^, A.T. Lely^3^, C.H.A. Nijboer^1^, F.E. Hoebeek^1^, F. Terstappen^1,3,5^

^1^Division of Women & Baby, department for Developmental Origins of Disease (DDOD), Brain Center UMC Utrecht, Wilhelmina Children’s Hospital, Utrecht University, Utrecht, the Netherlands

^2^Division of LAB, department CDL research, Nano medicine, University Medical Center Utrecht, Utrecht University, Utrecht, the Netherlands

^3^Division of Women & Baby, department of Obstetrics, Wilhelmina Children’s Hospital, Utrecht University, Utrecht University, Utrecht, the Netherlands

^4^Division of Internal Medicine and Dermatology, department of Nephrology and Hypertension, University Medical Center, Utrecht, the Netherlands.

^5^Division of Women & Baby, department of Neonatology, Wilhelmina Children’s Hospital, Utrecht University, Utrecht, the Netherlands

Correspondence:

*Fieke Terstappen (F.Terstappen@umcutrecht.nl)*

## Supplemental Methods

**Table S1. ARRIVE guideline checklist.**
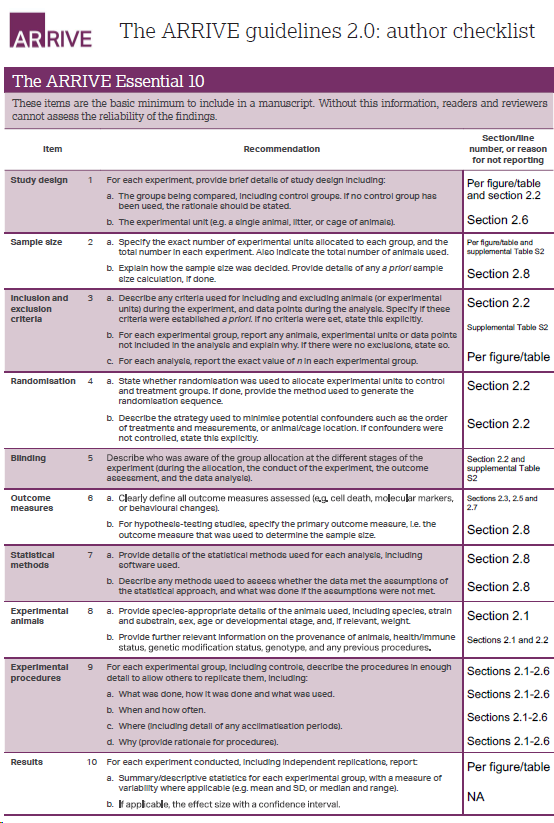


**Table S2. Overview of blinding of investigator during analysis and animal exclusions.**

|  | Blinding during the analysis | Images and analyses performed by | Included in analyses | Excluded from analyses | Reason |
| --- | --- | --- | --- | --- | --- |
| Brain (collection and processing): |  |  | 13 SHAM  16 RUPP | 0 |  |
| H&E (brain area and cortical thickness) | Yes | 1 researcher | 13 SHAM  16 RUPP | 0 |  |
| H&E (cerebral microbleeds) | Yes | 3 independent researchers | 13 SHAM  16 RUPP | 0 |  |
| laminin | Yes | 1 researcher | 13 SHAM  16 RUPP | 0 |  |
| albumin | Yes | 3 independent researchers | 13 SHAM  16 RUPP | 0 |  |
| Heart (collection and processing): |  |  | 9 SHAM  12 RUPP | 4 SHAM  4 RUPP | 8 hearts were lost during the embedding procedure. |
| H&E macroscopic | Yes | 2 independent researchers | LV:  8 SHAM  11 RUPP  RV:  9 SHAM  12 RUPP | LV:  1 SHAM  1 RUPP  RV:  0 SHAM  0 RUPP | 1 sham and one RUPP fetus excluded for LV due to inadequate staining. For RV there were no additional exclusions. |
| H&E microscopic | Yes | 1 researcher | LV + RV:  9 SHAM  12 RUPP | LV + RV:  0 SHAM  0 RUPP |  |
| Ki67 | Yes | 2 independent researchers | LV + RV:  8 SHAM  12 RUPP | LV + RV:  1 SHAM  0 RUPP | 1 sham fetus excluded for LV and RV due to inadequate staining |
| Laminin | Yes | 1 researcher | LV:  9 SHAM  12 RUPP  RV:  9 SHAM  9 RUPP | LV:  0 SHAM  0 RUPP  RV:  0 SHAM  3 RUPP | 3 RUPP fetuses excluded for RV due to inadequate staining |
| Kidney (collection and processing): |  |  | 12 SHAM  16 RUPP | 1 SHAM | 1 kidney (sham) |
| H&E | Yes |  | 12 SHAM  16 RUPP |  |  |
| JG12 | Yes |  | 12 SHAM  16 RUPP |  |  |
| Ki67 | Yes |  | 12 SHAM  16 RUPP |  |  |

Summary of blinding, number of researchers performing the analyses, inclusions, exclusions and the reasons of exclusion are listed per organ. No animals were excluded based on our *a priori* exclusion criteria that the surgery resulted in resorption of all fetuses.

**Protocol for scoring glomerular maturation in the rat**

The human scoring model for glomerular maturation (as described in Sutherland, 2011) fails to directly to extrapolate to rat models due to the simplicity of the renal maturation in rats. Therefore, we applied a simplified staging method consisting of three different stages (**Figure S1**). While the first stage for human glomerular maturation involves renal vesicles, we excluded this in the maturation scoring for rats since the identification of renal vesicle in rat tissue is difficult without specific markers. The comma- and S-shaped categories were merged into one to limit wrongly categorizing as a consequence of missing the lower limb due to sectioning. Additionally, the three mature stages have been condensed into one mature stage for the rat tissue.

Noteworthy, the presence or absence of a Bowman’s space does not form a good criterium as it highly depends on the cross section; when catching the juxtaglomerular apparatus, the glomerulus might appear as mature while actually still being in a developing stage. Therefore the best approach concerns focusing on the podocytes, as described in the characteristics in the table below.

When in doubt, we recommend using the location of the glomerulus to make one option more
likely. For example, when it is difficult to decide between the S and C stage, a location closer to
the capsule could be used as an argument to categorize the glomerulus as S.


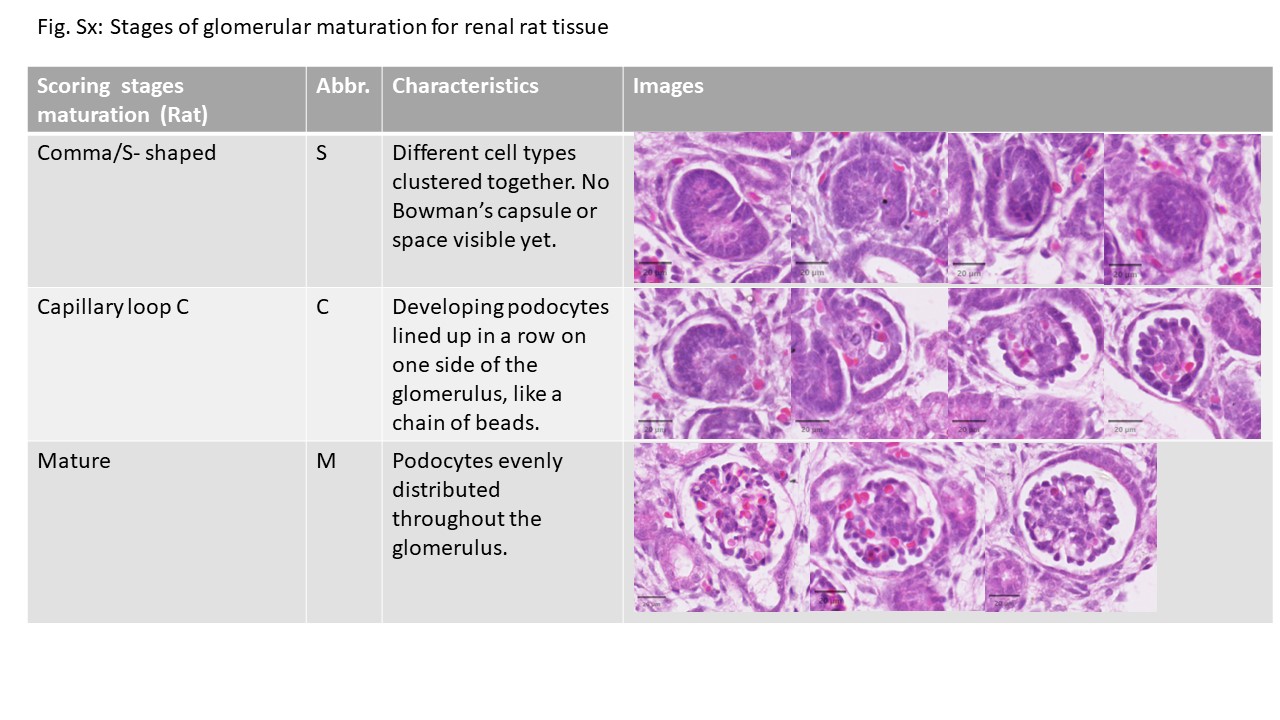


**Figure S1. Overview determination of the stages of maturation with illustration of comma/S-shaped (S) stage, capillary loop (C) and mature (M) stage.**

## Supplemental Results

**Table S3. Brain results presented by sex.**

| **Brain outcomes** | **Male** | | **Female** | | ***p*-value two-way ANOVA** | | |
| --- | --- | --- | --- | --- | --- | --- | --- |
|  | sham  (n=9) | RUPP  (n=8) | sham  (n=4) | RUPP  (n=7) | Group | Sex | Group × Sex |
| Brain area (cm^2^) | 0.16±0.02 | 0.15±0.03 | 0.16±0.03 | 0.14±0.01 | 0.07 | 0.96 | 0.62 |
| Total cortical thickness (mm) | 0.64±0.05 | 0.58±0.07 | 0.65±0.07 | 0.59±0.03 | **<0.01** | 0.65 | 0.73 |
| Cortical thickness A30 (mm) | 0.63±0.03 | 0.57±0.08 | 0.62±0.07 | 0.60±0.07 | 0.12 | 0.64 | 0.49 |
| Cortical thickness motor (mm) | 0.58±0.06 | 0.54±0.06 | 0.62±0.09 | 0.53±0.05 | 0.06 | 0.52 | 0.51 |
| Cortical thickness sensory (mm) | 0.70±0.09 | 0.65±0.05 | 0.72±0.08 | 0.63±0.04 | **0.01** | 0.91 | 0.51 |
| Total microbleeds (n)/brain area (cm) | 31.93±32.5 | 31.33±26 | 17.74±14.1 | 38.08±48.7 | 0.47 | 0.79 | 0.45 |
| Total albumin leakages (n)/brain area (cm) | 13.79±10.2 | 6.82±5.5 | 20.84±25.7 | 4.94±6.5 | **0.02** | 0.58 | 0.35 |
| Total laminin (%)/brain area (cm) | 14.15±5.1 | 16.61±3.8 | 14.75±5.5 | 15.66±5.5 | 0.40 | 0.93 | 0.69 |
| Laminin left A30 (%)/brain area (cm) | 14.4±4.7 | 19.04±4.5 | 16.72±9.2 | 18.65±5.8 | 0.16 | 0.67 | 0.55 |

Data are shown as mean ± SD. The sex of one RUPP fetus could not be determined due to not enough tissue available. Post-hoc analysis for sex was not performed considering no statistical difference for sex or the interaction (group x sex) derived from the ANOVA analysis. n, amount; RUPP, reduced uterine perfusion pressure.

**Table S4. Heart results presented by sex.**

| **Heart outcomes** | **Male** | | **Female** | | ***p*-value two-way ANOVA** | | | ***p*-value post-hoc** | |
| --- | --- | --- | --- | --- | --- | --- | --- | --- | --- |
|  | sham  (n=5-6) | RUPP  (n=4-5) | sham  (n=3) | RUPP  (n=4-6) | Group | Sex | Group × Sex | Male | Female |
| LV wall thickness (μm)/fetal weight (g) | 35.1±16.0 | 48.2±9.4 | 41.6±4.5 | 40.5±7.2 | 0.25 | 0.91 | 0.25 | NA | NA |
| RV wall thickness (μm)/fetal weight (g) | 29.3±8.2 | 46.8±23.3 | 26.2±7.4 | 31.2±8.4 | 0.09 | 0.16 | 0.34 | NA | NA |
| LV nuclei per field (n) | 222.6±31.4 | 219.4±52.7 | 216.5±16.5 | 240.3±34.9 | 0.57 | 0.68 | 0.45 | NA | NA |
| RV nuclei per field (n) | 218.2±40.3 | 213.8±40.4 | 216.9±11.7 | 231.8±39.6 | 0.77 | 0.64 | 0.59 | NA | NA |
| LV Ki67 cells per field (n) | 36.7±18.0 | 55.0±30.6 | 38.9±41.0 | 47.0±18.4 | 0.3 | 0.81 | 0.68 | NA | NA |
| RV ki67 cells per field (n) | 45.4±19.3 | 40.7±17.6 | 43.0±35.3 | 40.2±16.9 | 0.71 | 0.89 | 0.92 | NA | NA |
| LV laminin area per field (%) | 12.6±4.6 | 25.8±10.9 | 69.5±58.1 | 29.5±18.9 | 0.25 | **0.01** | **0.03** | 0.61 | 0.06 |
| RV laminin area per field (%) | 22.9±27.6 | 22.5±16.4 | 44.1±26.5 | 46.4±31.5 | 0.94 | 0.11 | 0.92 | NA | NA |

Data are shown as mean±SD. Eight hearts were lost during the embedding procedure (See Table S2). The sex of one RUPP fetus could not be determined due to a lack of tissue available. One sham male was excluded from LV wall thickness and LV ki67 cells per field due to inadequate stainings, as well as one RUPP male, that was excluded from % RV laminin area per field due to inadequate staining. As for the females, two RUPP were excluded for % RV laminin area per field due to inadequate staining. Post-hoc analysis for sex was only performed in case of statistical difference for sex or the interaction (group x sex) derived from the ANOVA analysis. LV, left ventricle; n, amount; NA; not applicable; RUPP, reduced uterine perfusion pressure; RV, right ventricle.

**Table S5. Kidney results presented by sex.**

| **Kidney outcomes** | **Male** | | | **Female** | | ***p*-value two-way ANOVA** | | |
| --- | --- | --- | --- | --- | --- | --- | --- | --- |
|  | sham  (n=9) | RUPP  (n=8) | sham  (n=3) | | RUPP  (n=7) | Group | Sex | Group × Sex |
| Total number of glomeruli (n) | 39.9±12.2 | 43.6±10.04 | 32.7±13.6 | | 36.3±15.2 | 0.50 | 0.18 | 0.99 |
| S-shape glomeruli (%) | 29.7±27.4 | 17.3±14.34 | 19.4±6.9 | | 46.8±27.6 | 0.45 | 0.33 | 0.05 |
| Capillary loop glomeruli (%) | 35.8±17.5 | 43.3±16.06 | 42.5±7.2 | | 33.6±9.8 | 0.91 | 0.8 | 0.20 |
| Mature glomeruli (%) | 34.9±19.8 | 39.4±21.32 | 38.1±14.0 | | 31.4±20.8 | 0.9 | 0.78 | 0.51 |
| Average of glomerular area (μm^2^) | 2798.4±692.2 | 2825.7±500.51 | 3097.3±275.0 | | 2510.5±810.7 | 0.32 | 0.98 | 0.27 |
| Glomerular area S-shape (μm^2^) | 2671.4±673.3 | 2768.3±643.23 | 2934.4±181.5 | | 2286.5±788.0 | 0.34 | 0.70 | 0.20 |
| Glomerular area capillary loop (μm^2^) | 2747.5±603.7 | 2797.8±483.88 | 3069.1±227.6 | | 2653.0±697.3 | 0.46 | 0.72 | 0.35 |
| Glomerular area mature (μm^2^) | 3024.6±1039.1 | 2730.3±806.91 | 3173.0±407.6 | | 2655.4±925.2 | 0.31 | 0.93 | 0.78 |
| Glomerular density (μm^2^) | 22.0±7.2 | 22.8±4.72 | 17.8±3.6 | | 28.2±13.3 | 0.13 | 0.87 | 0.19 |
| Ki67 cells/kidney area (n) | 1.0±1.0 | 1.1±1.00 | 1.0±0.6 | | 1.±1.3 | 0.69 | 0.86 | 0.93 |
| JG12 cells/kidney area (n) | 0.9±0.3 | 0.9±0.6 | 1.3±0.8 | | 1.0±0.3 | 0.56 | 0.29 | 0.45 |

Data are shown as mean±SD. One kidney could not be used for analysis (see Table S2). The sex of one RUPP fetus could not be determined due to a lack of tissue available. n, amount; RUPP, reduced uterine perfusion pressure.

**Table S6. Glomerular diameter, density, and maturation in E19 fetuses.**

|  | **sham (n=12)** | **RUPP (n=16)** | ***p*-value** |
| --- | --- | --- | --- |
| Total glomerular diameter (μm) | 2873.1±616.8 | 2674.9±636.2 | 0.41 |
| Total glomerular number (n) | 39.0±12.5 | 39.4±12.8 | 0.94 |
| Total glomerular density (per mm^2^) | 20.95±6.6 | 22.69±9.7 | 0.44 |
| Glomerular S-stage (%) | 28.0±23.7 | 31.0±24.9 | 0.75 |
| Glomerular C-stage (%) | 37.5±15.5 | 38.2±13.7 | 0.89 |
| Glomerular M-stage (%) | 38.1±14.4 | 38.4±18.3 | 0.97 |

Data are shown as mean±SD. C-stage, capillary loop stage; E, embryonic day; M-stage, mature stage; n, amount; RUPP, reduced uterine perfusion pressure; S-stage, comma/S-shaped stage.


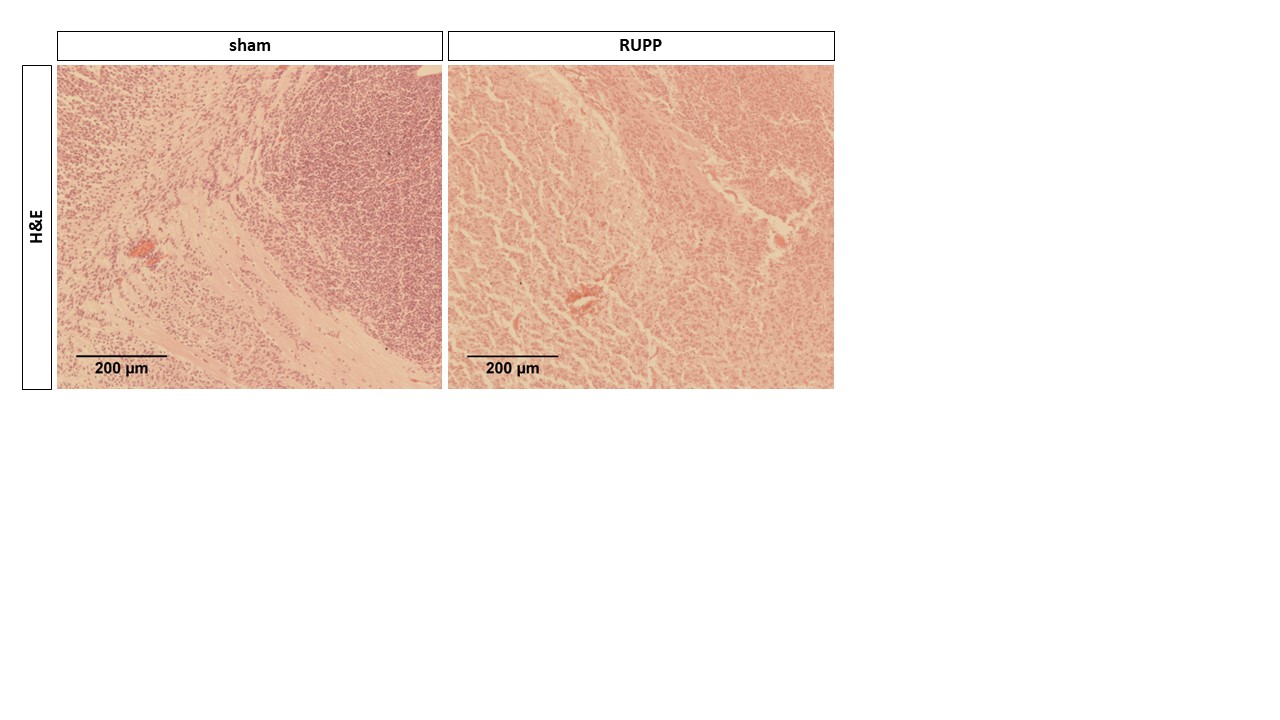


**Figure S2. Microscopic examples of cerebral microbleeds stained with H&E in sham and RUPP at E19.** E19, embryonic day 19; H&E, hematoxylin and eosin; RUPP, reduced uterine perfusion pressure.


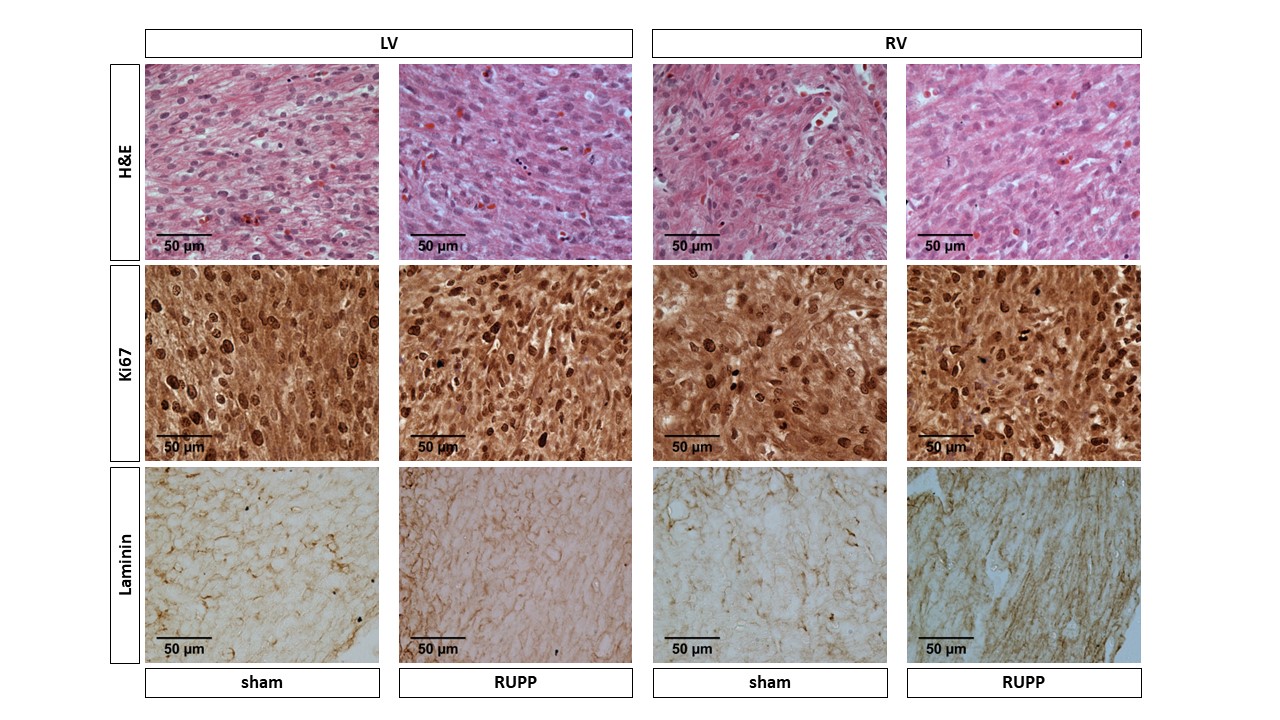


**Figure S3. Microscopic examples of H&E, Ki67 and laminin stainings of LV and RV of the heart of both sham and RUPP groups at E19.** E19, embryonic day 19; H&E, hematoxylin and eosin; LV, left ventricle; RUPP, reduced uterine perfusion pressure; RV, right ventricle.

**
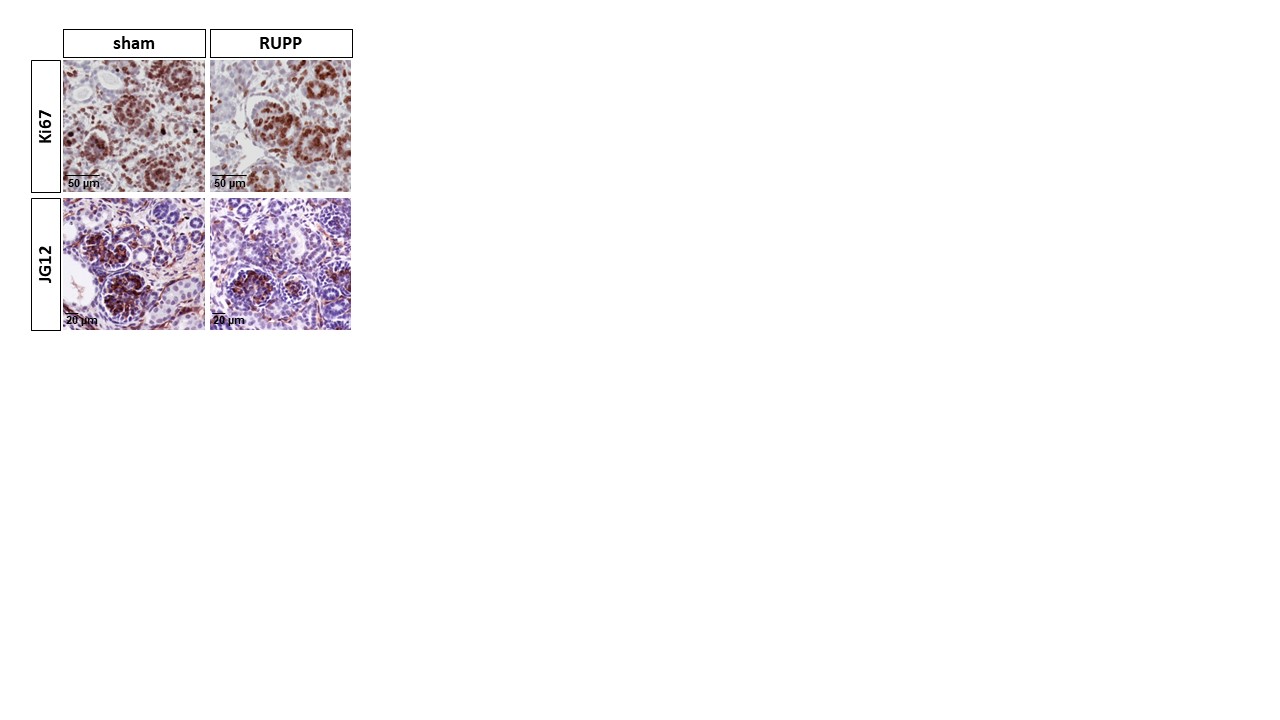
**

**Figure S4. Microscopic examples of Ki67 and JG12 stainings of the kidney of both sham and RUPP groups at E19.** E19, embryonic day 19; RUPP, reduced uterine perfusion pressure.
